# Design of an Afocal Telescope System Integrated with Digital Imaging for Enhanced Optical Performance

**DOI:** 10.3390/mi17010062

**Published:** 2025-12-31

**Authors:** Yi-Lun Su, Wen-Shing Sun, Chuen-Lin Tien, Yen-Cheng Lin, Yi-Hong Liu

**Affiliations:** 1 Department of Optics and Photonics, National Central University, Chungli 32001, Taiwan; a0357841@gmail.com (Y.-L.S.); wssun@dop.ncu.edu.tw (W.-S.S.); 111240003@cc.ncu.edu.tw (Y.-C.L.); yihongluis@gmail.com (Y.-H.L.); 2 Department of Electrical Engineering, Feng Chia University, Taichung 40724, Taiwan

**Keywords:** afocal telescope, digital imaging, RMS wavefront error, eyepiece design, pupil matching, modulation transfer function

## Abstract

This study presents the design and optimization of a digital-imaging afocal telescope system that integrates an afocal telescope architecture with an imaging optical subsystem. The proposed system employs a combination of spherical and aspherical optical elements to enhance imaging flexibility, reduce aberrations, and ensure effective system coupling. Proper pupil matching is achieved by aligning the exit pupil of the afocal telescope with the entrance pupil of the imaging system, ensuring minimal vignetting and optimal energy transfer. Circular apertures and lens elements are used throughout the system to simplify alignment and minimize pupil-matching errors. The complete system comprises three imaging optical subsystems and a digital camera module, each independently optimized to ensure balanced optical performance. The design achieves an overall magnification of 16×, with near-diffraction-limited quality confirmed by an RMS wavefront error of 0.0474λ and a Strehl ratio of 0.915. The modulation transfer function (MTF) reaches 0.42 at 80 lp/mm, while the distortion remains below 4.87%. Chromatic performance is well controlled, with maximum lateral color deviations of 1.007 µm (short-to-long wavelength) and 1.52 µm (short-to-reference wavelength), evaluated at 656 nm, 587 nm, and 486 nm. The results demonstrate that the proposed digital-imaging afocal telescope system provides high-resolution, low-aberration imaging suitable for precision optical applications.

## 1. Introduction

An afocal optical system refers to an optical configuration in which the light beam exhibits no net convergence or divergence, meaning the system has an infinite effective focal length [1]. The simplest form of such a system consists of two optical elements separated by a distance equal to the sum of their focal lengths (d = f_1_ + f_2_). The main advantages of an afocal telescope include focus-free operation, portability, and ease of use.

This study adopts an afocal telescope architecture combining spherical and aspherical optical elements, emphasizing the integration and alignment of individual optical subsystems. To ensure proper coupling between the afocal telescope and the imaging optical system, the exit pupil of the telescope must be precisely aligned with the entrance pupil of the imaging optics. Therefore, the exit pupil must match the pupil geometry of the eyepiece, erecting mirror, and objective lens to maintain stable imaging performance. Addressing this pupil alignment issue remains a critical challenge in off-axis afocal telescope architectures. To overcome this, a coaxial optical design is adopted in this study to preserve pupil quality, and the root mean square (RMS) wavefront error is used as a key metric for evaluating final image quality. Classic telescope designs such as the Schmidt–Cassegrain and Maksutov–Cassegrain configurations were considered in this research. However, in these designs, the secondary mirror is positioned in front of the primary mirror, which introduces central obscuration and reduces the effective light throughput. Therefore, the size of the secondary mirror aperture must be carefully considered to minimize light loss and its impact on imaging performance [2]. These catadioptric designs have successfully demonstrated how long optical paths can be folded into compact configurations for astronomical use. Traditional catadioptric telescopes, which combine reflective and refractive elements, achieve both high optical performance and compactness [3,4]. Nonetheless, they still face challenges related to aberration correction, thermal stability, and manufacturing cost.

In 2019, Galan [5] developed a compact refractive imaging optical architecture, although the design suffered from system obscuration caused by the reflective surface, which degraded diffraction-limited performance with low light throughput. In 2021, Jahromi [6,7,8] and Kim [9] proposed afocal telescope systems designed for rifle-mounted applications, presenting concepts of angular magnification between the objective and eyepiece but without providing optical design methodologies. In 2021, Zhang et al. [10] introduced a Newton-type afocal optical system integrated with an imaging system, but it faced alignment challenges and diffraction degradation due to secondary mirror obscuration. For off-axis afocal systems, Bauer et al. [11] in 2023 proposed a design framework based on freeform surfaces, focusing on analyzing and controlling exit pupil alignment; the design resulted in a maximum RMS wavefront error below <0.07 λ (λ = 632.8 nm). More recently, in 2024, Zou et al. [12] presented a 5× zoom afocal telescope using a deformable mirror. To correct asymmetric aberrations, an XY-polynomial surface was applied to the primary mirror along with a folding plane mirror. Simulation results demonstrated a 5× zoom capability with a maximum RMS wavefront error below 0.125 λ (λ = 632.8 nm).

None of the previous studies have integrated an afocal telescope system with an imaging system into a unified optical design. In an afocal configuration, light enters through the objective lens and exits the eyepiece as parallel rays. This study aims to optimize such configurations to achieve superior optical performance and imaging quality through an afocal optical architecture. We propose a digital-imaging afocal telescope, which combines an afocal telescope system with an imaging optical system to form a coaxial optical architecture capable of continuous long-distance image capture and recording. This design effectively reduces RMS wavefront error and chromatic aberration, thereby improving overall image quality. This system is suitable for visual observation applications, such as bird watching, whale watching, and large-scale sporting event monitoring. Furthermore, by integrating a digital-imaging module (such as a smartphone camera) positioned beyond the eye relief, it minimizes visual fatigue during extended observation through the eyepiece, enhancing both user comfort and operational convenience.

We propose a novel digital-imaging afocal telescope design, in which the overall optical configuration incorporates two reflective mirrors positioned between the objective lens and the erecting optics to form a compact folded optical path. This configuration offers the advantages of structural compactness and high-resolution imaging capability.

## 2. Design Methodology

At the initial stage of this study, various types of telescope optical designs were collected and analyzed. Based on these analyses, the proposed design concept was incorporated into the optical system research [13,14]. The telescope and digital camera were combined and independently designed and optimized using the imaging optical design software CODE V (2024.03). In the telescope system, each subsystem—such as the objective lens and eyepiece optical systems—was developed as an independent imaging unit. After completing the individual designs, the eyepiece system was reversed and integrated with the objective lens and erecting lens systems. Subsequently, the two subsystems were aligned at a common focal point in CODE V to form an afocal optical configuration. Optical analyses were then conducted to verify the final imaging performance.

In this study, the aperture stop (STOP) of each optical system was placed on its first optical surface, so the entrance pupil of each system coincided with that surface. This configuration ensured that light could properly enter each subsystem and allowed the calculation of both object-side and image-side numerical apertures (NAs) for each telescope subsystem, achieving precise alignment and integration between the afocal telescope and the imaging subsystems. After assembling all telescope subsystems into a complete afocal telescope configuration, optical performance analyses were performed. If the results did not meet the design criteria, individual subsystems were re-optimized before reintegration.

**Figure 1:**
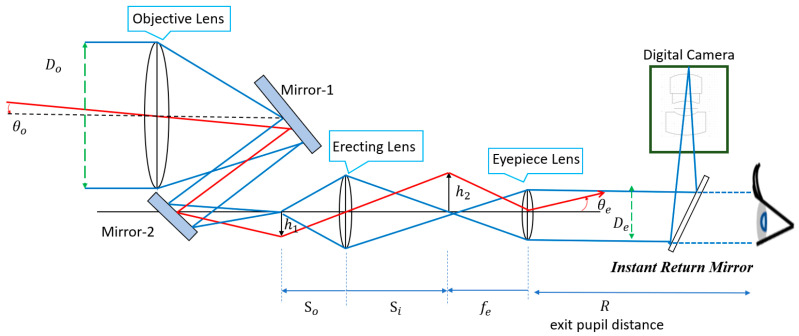
The layout of the digital-imaging afocal telescope system.

In this optical configuration, small-angle obliquely incident parallel light from a distant object passes through the objective lens and is transformed into large-angle oblique parallel light, which propagates toward the observer [15,16]. Figure 1 illustrates the optical configuration of the proposed digital-imaging afocal telescope system. A movable mirror is placed within the eye-relief region of the telescope. When the system is calibrated for direct visual observation, the mirror is retracted from the optical path, allowing the observer to view the angularly magnified telescope image directly. When image recording with a digital camera is required, the mirror is inserted into the eye-relief region at a 45° tilt, redirecting the angularly magnified image from the telescope toward the digital camera for image capture and storage. The red line represents the off-axis chief ray, which enters obliquely from a distant object, passes through the center of the aperture stop, and reaches the edge of the image plane. The blue line represents the on-axis (0° field) marginal ray, which is parallel light incident from the 0° field, touches the edge of the aperture stop, and finally reaches the center of the image plane.

By integrating the objective lens, erecting lens, and eyepiece subsystems, the complete optical path of the telescope can be established. Through optical analysis, it can be derived that the angular magnification M*_P_* of the afocal telescope system is equal to the ratio of the entrance pupil diameter to the exit pupil diameter. This relationship can also be expressed as the negative product of the transverse magnification M*_tr_* of the erecting mirror system and the ratio of the image focal length of the objective lens to that of the eyepiece, as shown in Equation (1) [17,18].

(1) $$M_{p}=-M_{tr}\frac{f_{o}^{\prime }}{f_{e}}$$

where M*_P_* is the angular magnification of the afocal telescope system; M*_tr_* is the transverse magnification of the erecting mirror system; $f_{o}^{\prime }$ is the focal length of the objective lens; $f_{e}$ is the focal length of the eyepiece lens.

The angular magnification (M*_p_*) of the afocal telescope system can also be expressed as the ratio of the tangent function value of the incident angle to the tangent function value of the exit angle. The formula is rewritten as follows in Equation (2) [18].

(2) $$M_{p}=\frac{tan\theta_{e}}{tan\theta_{o}}=\frac{-\frac{h_{2}}{f_{e}^{\prime }}}{\frac{h_{1}}{f_{o}^{\prime }}}=-\frac{f_{o}^{\prime }}{f_{e}^{\prime }}\times \frac{h_{2}}{h_{1}}=-\frac{f_{o}^{\prime }}{f_{e}^{\prime }}\times {Mt}_{r}=\frac{D_{o}}{D_{e}}$$

where *θ_o_* is the incident angle of the object lens and *θ_e_* is the exit angle of the eyepiece lens. *h*_1_ is the image height of the object lens; *h*_2_ is the image height of the erecting lens and it is also the object height of the eyepiece lens; $f_{o}^{\prime }$ is the focal length of objective lens; $f_{e}$ is the focal length of the eyepiece lens. $D_{o}$ is the entrance pupil diameter; $D_{e}$ is the exit pupil diameter.

### 2.1. Objective Lens Design

**Figure 2:**
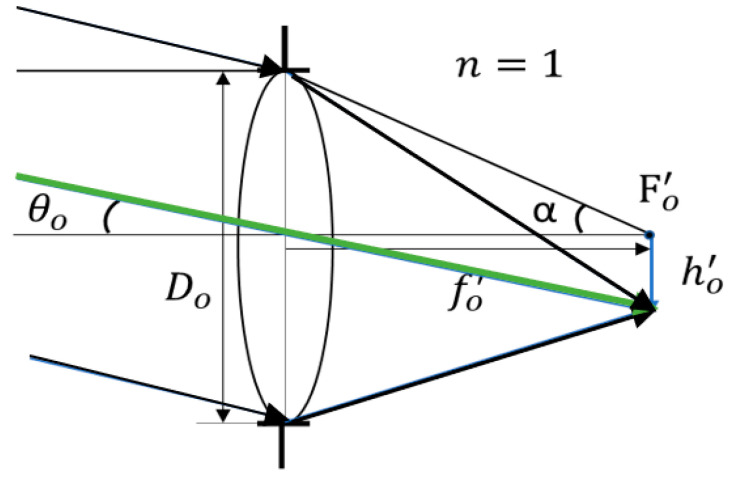
Objective system design and symbol definition.

The objective lens is the first optical lens group that receives incoming light. Its aperture is positioned at the point in the optical system where the light obstruction is the greatest. The aperture size directly affects the brightness of the incoming light in the optical system [19]. In this design, the aperture is placed on the first surface of the objective lens, meaning that its diameter determines the amount of incoming light and indirectly influences the image brightness. The objective lens system is shown in Figure 2 and the relationship is shown in Equation (3).

(3) $$tan\theta_{o}=\frac{{h^{\prime }}_{o}}{{f^{\prime }}_{o}}$$

where $D_{o}$ is the entrance pupil diameter of the objective lens; $\theta_{o}$ is incident angle of the principal ray in object; $h_{o}^{\prime }$ is image height; $f_{o}^{\prime }$ is the focal lens of the objective lens; α is incident angle of the marginal ray in object space; ${NA}_{o}^{\prime }$ = sinα.

### 2.2. Erecting Lens Design

**Figure 3:**
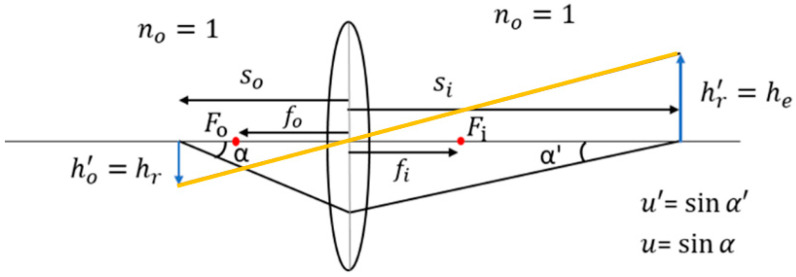
Erecting lens design and symbol definition.

The objective lens produces an inverted image on the image plane, where both the vertical and horizontal orientations are reversed [20]. To ensure that the object’s orientation matches the final image, an erecting lens is introduced between the objective lens and the eyepiece, as shown in Figure 3. Lateral magnification (${Mt}_{r})\;$ of the erecting lens [21,22] is given in Equation (4).

(4) $${Mt}_{r}=\frac{s_{i}}{s_{o}}=\frac{nu}{n^{\prime }u\prime }=\frac{{NA}_{r}}{{NA}_{r}^{\prime }}=\frac{h_{r}^{\prime }}{h_{r}}$$

where $h_{r}^{\prime }$ is the paraxial height of the erecting lens; $h_{r}$ represents the object height of the erecting lens; ${NA}_{r}\prime$ is numerical aperture of the erecting lens in an image space; ${NA}_{r}$ is numerical aperture of the erecting lens in object space.

### 2.3. Eyepiece Lens Design

**Figure 4:**
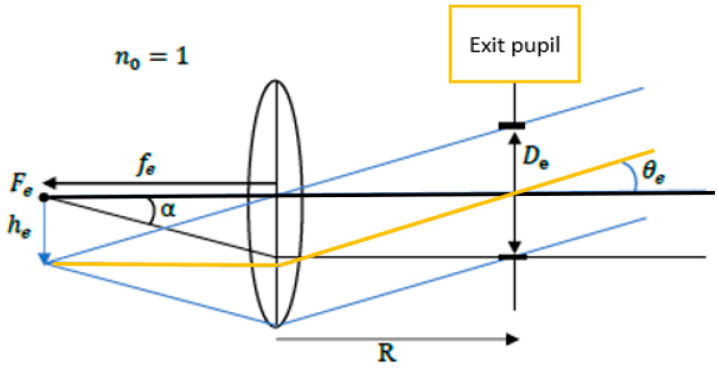
Eyepiece lens design and symbol definition.

The eyepiece lens constitutes the final optical assembly in the proposed telescope system, facilitating the exit of parallel light from the optical system [23,24]. Its structural configuration is illustrated in Figure 4. Since the overall system is designed as a confocal system, the eyepiece lens is designed in reverse and optimized by the optical design software to ensure proper alignment and performance.

Where $f_{e}$ is the focal lens of the eyepiece; $D_{e}$ is the size of the exit pupil; $h_{e}$ is the image height of the eyepiece lens and $\theta_{e}$ is the angle of the principal ray in the image; R is the distance between the eyepiece lens and the exit pupil.

### 2.4. Optical Design of the Digital-Imaging Afocal Telescope System

The initial specifications of the digital-imaging afocal telescope system include an objective lens entrance pupil diameter of 40 mm and an eyepiece exit pupil size of 2.5 mm. An angular magnification of 16× is achieved, as shown in Table 1, and design requirements are shown in Table 2. Tolerances [18] include the radius of curvature (DLF), the cylinder irregularity (CYD, CYN), the thickness (DLT), the refractive index (DLN), the V-number (DLV), the wedge (TRX, TRY), and the tilt (BTX, BTY). The range of tolerance parameters set for this lens design is shown in Table 3 [25].

### 2.5. Digital Camera System

The final stage of this optical system incorporates a digital camera, as illustrated in Figure 1. The primary function of this camera is to capture the optical image from the telescope and convert it into a digital signal. For this study, the digital camera system utilizes an Omni Vision OV5695 CMOS sensor [26] with a 5-megapixel sensing element. Its width, height, and diagonal length determine the effective sensor area. In this design, the image height is 2.30 mm. The detailed sensor specifications are listed in Table 4.

## 3. Optical Design Results

### 3.1. Objective Lens Design

**Figure 5:**
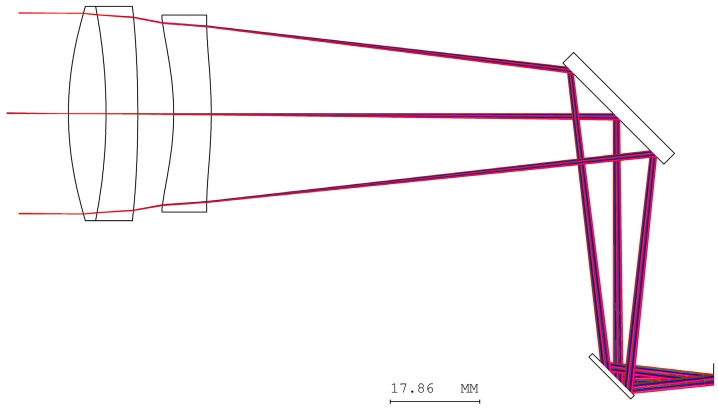
The layout for the objective lens design.

**Figure 6:**
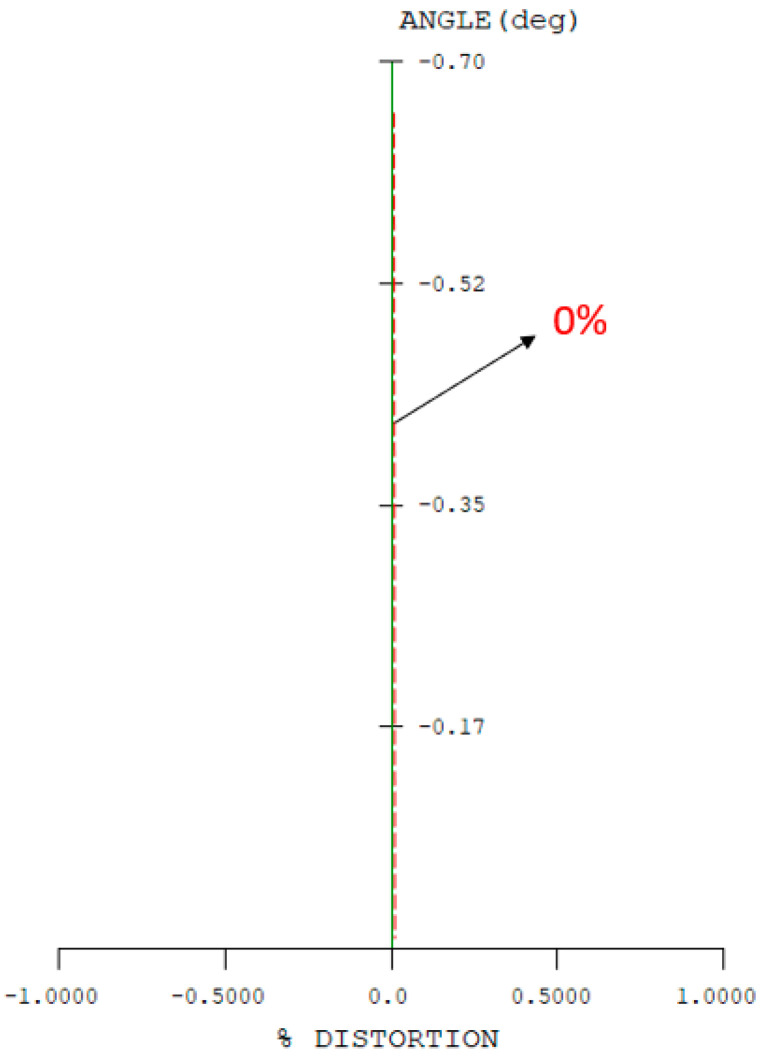
Optical distortion of the objective lens.

**Figure 7:**
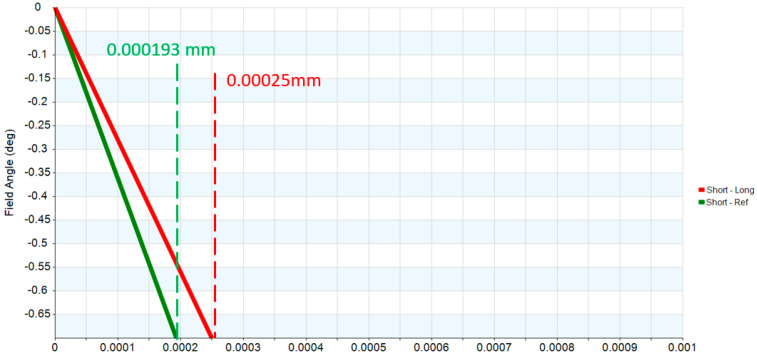
Lateral color of the objective lens.

**Figure 8:**
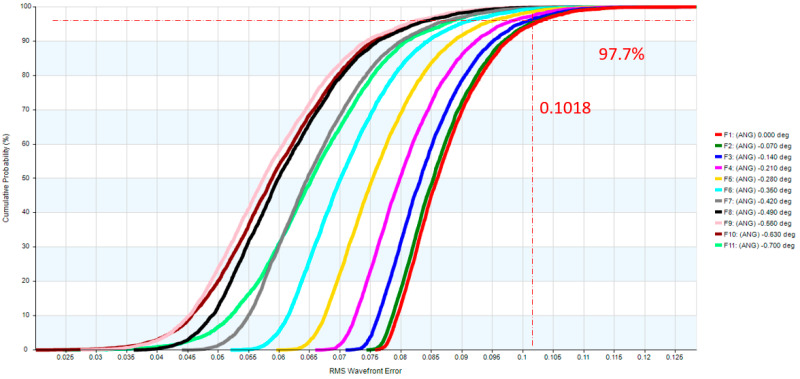
The tolerance analysis for the objective lens.

Two reflective mirrors are strategically positioned near the back focal plane, forming a catadioptric optical system that efficiently folds the optical path and significantly reduces the overall system length, as illustrated in Figure 5. The STOP is positioned on the first surface of the objective lens. The detailed design parameters of the objective lens are presented in Table 5. The negative sign along the vertical axis indicates the orientation of the mirror surfaces. The objective lens system exhibits zero distortion, and its lateral chromatic aberration is 0.25 µm (RED-short-long), as illustrated in Figure 6 and Figure 7, and the tolerance analysis at the 0 to 1.00 field position is shown in Figure 8. Data are shown in Table 6.

### 3.2. Erecting Lens Design

**Figure 9:**
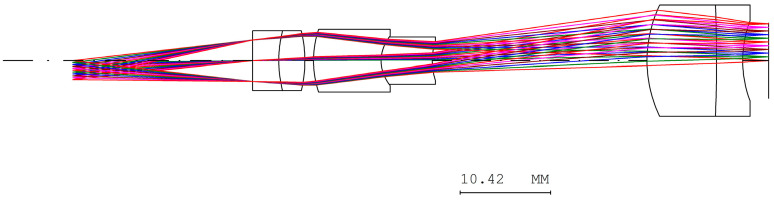
Lens layout of the erecting lens design.

**Figure 10:**
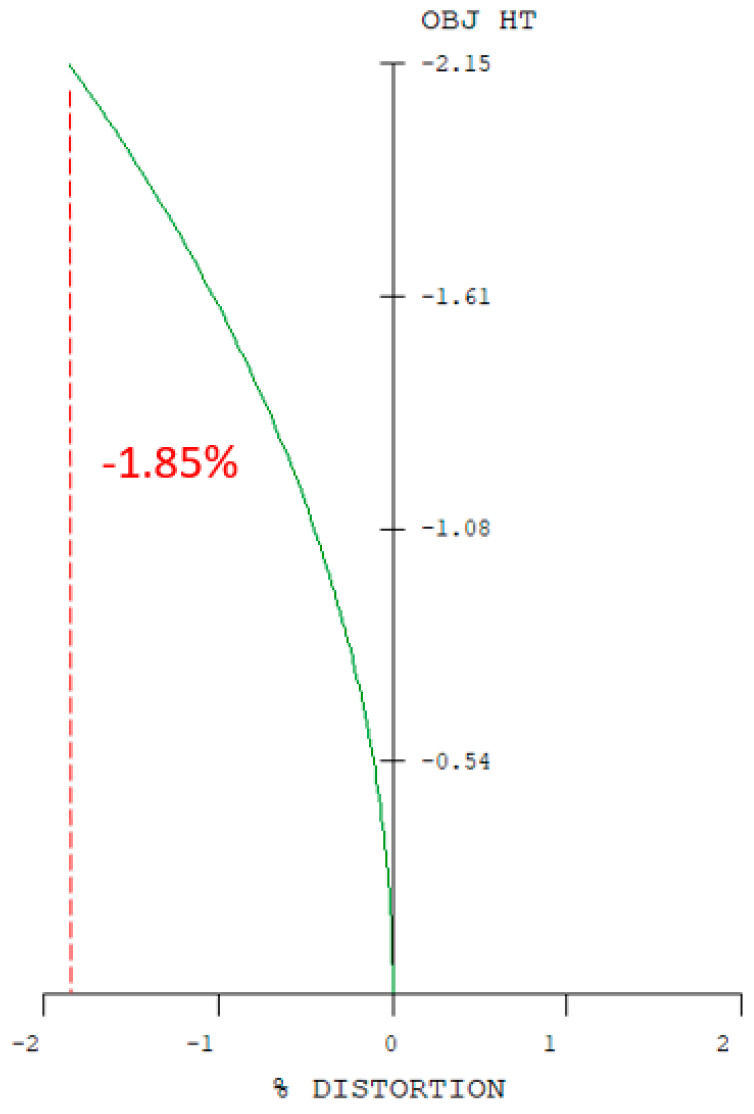
Distortion of the erecting lens.

**Figure 11:**
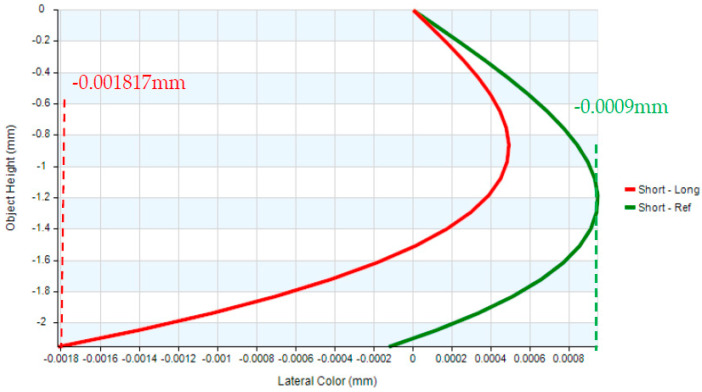
Lateral color of the erecting lens.

**Figure 12:**
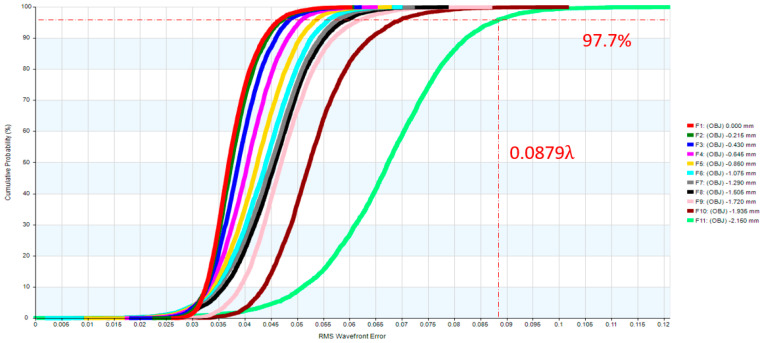
The tolerance analysis for the erecting lens.

In the erecting lens system, a stop was placed at the first surface of the optical system. The optical design parameters of the erecting lens and lens layout are provided in Table 7 and Figure 9. The distortion of the erecting lens system is −1.85%, and the lateral chromatic aberration is 1.817 µm (RED-short-long), as illustrated in Figure 10 and Figure 11. The tolerance analysis at the 0 to 1.00 field position is shown in Figure 12. Data are shown in Table 8.

### 3.3. Eyepiece Lens Design

**Figure 13:**
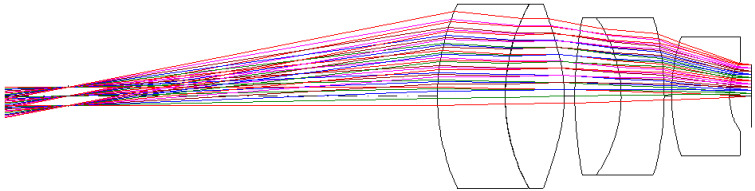
Lens layout for the eyepiece lens design.

**Figure 14:**
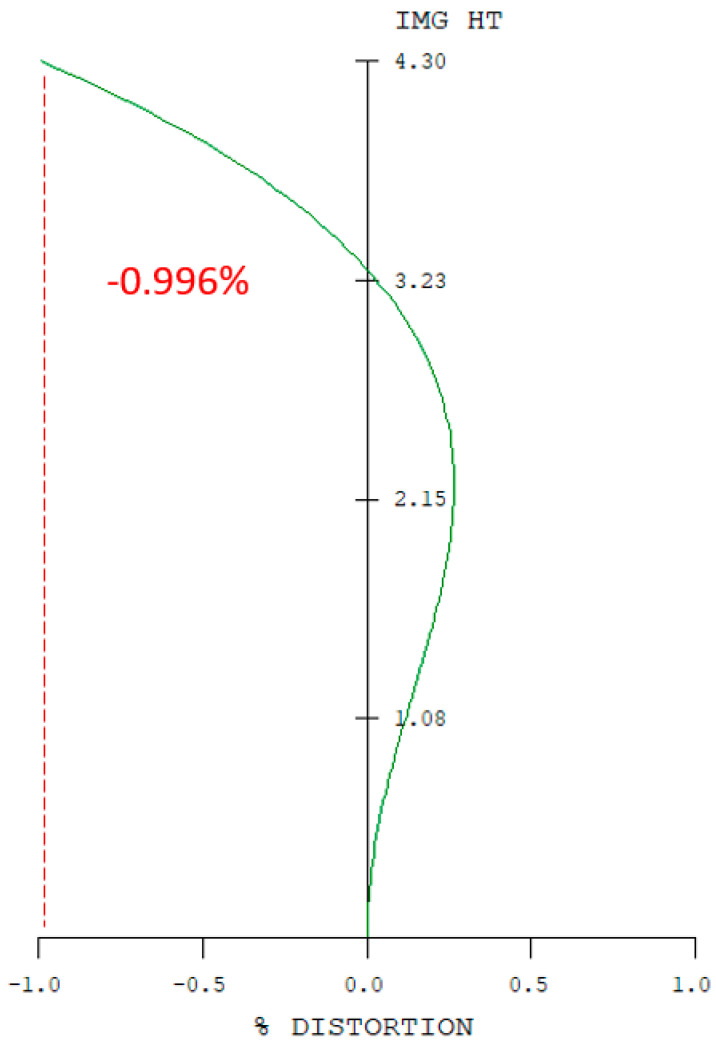
Distortion of the eyepiece lens.

**Figure 15:**
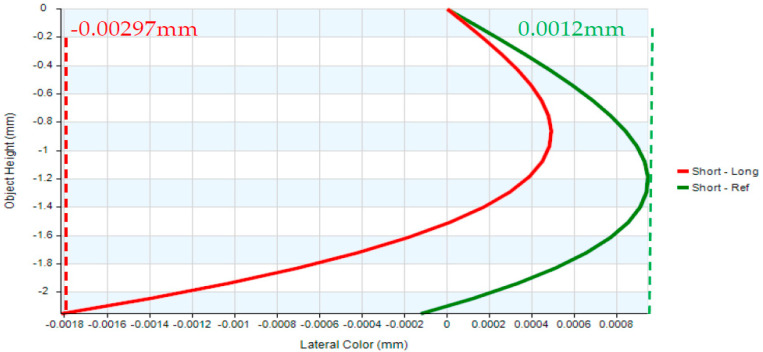
Lateral color of the eyepiece lens.

**Figure 16:**
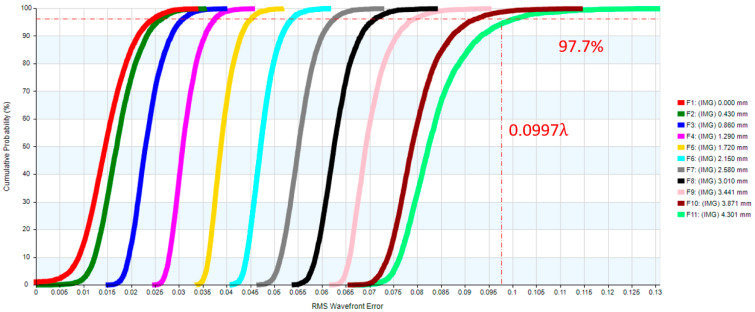
The tolerance analysis for the eyepiece lens.

The eyepiece structure of the digital-imaging afocal telescope system which requires reverse engineering during the design process using CODE V. The eyepiece lens layout is shown in Figure 13, and the optical design parameters of the eyepiece lens are listed in Table 9. The design results show that the distortion of the erecting lens is −0.99%, and the lateral chromatic aberration is 2.97 µm (RED-short-long). The distortion is illustrated in Figure 14, while the lateral chromatic aberration is shown in Figure 15. The tolerance analysis at the 0 to1.00 field position is shown in Figure 16, and tolerance analysis data are shown in Table 10.

### 3.4. Digital Camera System

In the final stage of the system design, a dedicated digital camera module was developed to capture and store images from the afocal telescope system, enabling simultaneous target observation and image recording. The camera integrates a 5-megapixel CMOS image sensor manufactured by OmniVision.

**Figure 17:**
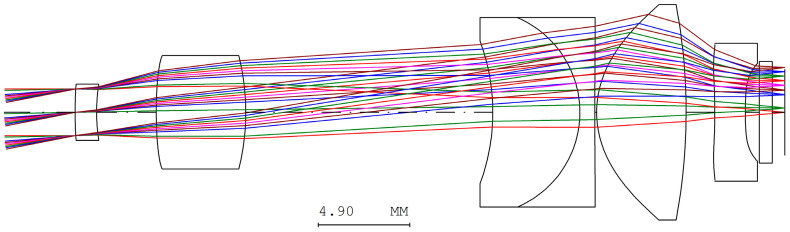
The lens layout for the digital camera system.

**Figure 18:**
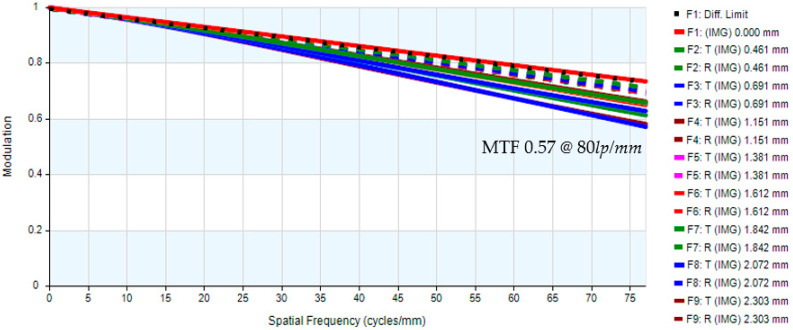
MTF of the digital camera system.

The sensor’s diagonal length measures 4.605 mm and is referenced against the eyepiece system’s half-field angle of 11.061°. The aperture stop of the digital camera is aligned with the exit pupil of the eyepiece system, which has a diameter of 2.5 mm. The preliminary specifications of the digital camera system are shown in Table 11, while the lens layout is illustrated in Figure 17. The optical design parameters of the digital camera system are shown in Table 12. The system is designed to achieve an MTF exceeding 0.5 at a spatial frequency of 80 lp/mm, as depicted in Figure 18.

**Figure 19:**
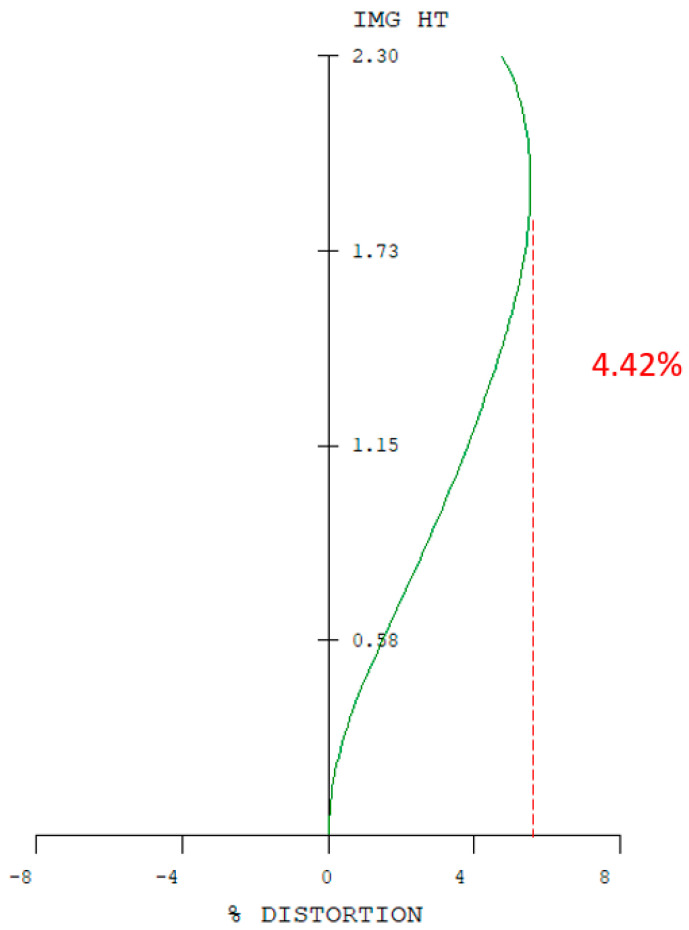
Distortion of the digital camera system.

**Figure 20:**
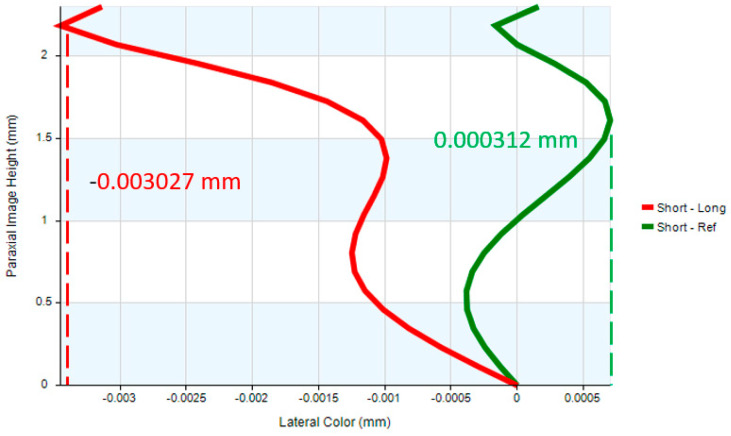
Lateral color of the digital camera system.

**Figure 21:**
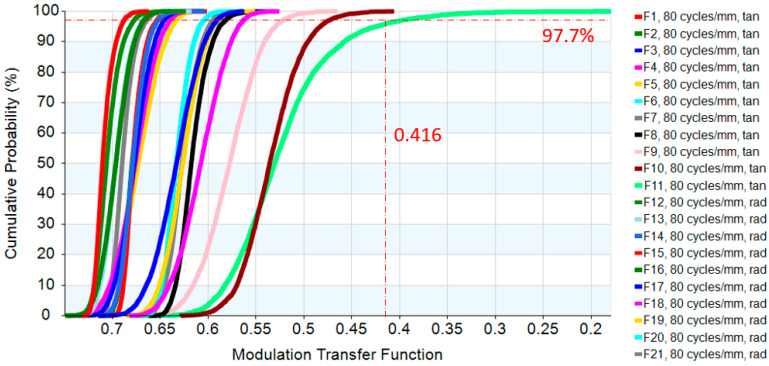
The tolerance analysis for the digital camera system.

The system’s image quality is also validated by analyzing lateral chromatic aberration, optical distortion, and other key performance metrics. The optical distortion remains below 4.42%, as illustrated in Figure 19. The maximum lateral chromatic aberration of the digital camera system occurs at a 0.95 field height, with a value of −3.12 µm (GREEN short-Ref), while the pixel size is 1.4 µm, as shown in Figure 19 and Figure 20. The tolerances at the 0 to 1.00 field position including tangential and radial values are shown in Figure 21. The tolerance analysis data are shown in Table 13.

## 4. Optical System Quality and Analysis of the Digital-Imaging Afocal Telescope System

The optical system designed in this study employs the Strehl ratio to evaluate imaging performance. A lower Strehl ratio indicates relatively poorer image quality. In practical applications, we set the requirement that the root mean square optical path difference (RMS OPD) must be less than 0.05 λ. Consequently, a Strehl ratio exceeding 0.8 indicates a high-quality optical system [23].

### 4.1. Afocal Telescope System

**Figure 22:**
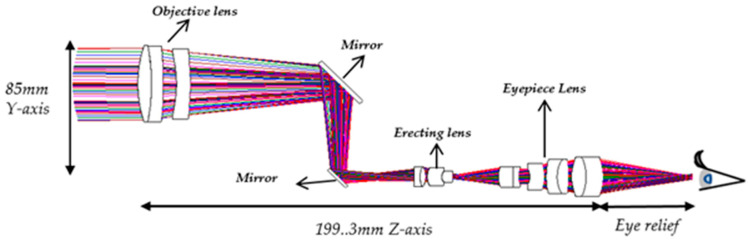
Optical layout for the afocal telescope system.

After completing the optical design, all subsystems were assembled into an integrated configuration. The image height produced by the objective lens serves as the object height for the erecting lens, which in turn generates an inverted image that matches the object height of the eyepiece. The total optical system length is 199.3 mm, providing an exit pupil distance (eye relief distance) of 50 mm. The final afocal telescope system configuration is illustrated in Figure 22. The overall system ultimately achieves an RMS wavefront error of 0.0474 λ and a Strehl ratio of 0.915, which are both below the predefined optical quality threshold, as summarized in Table 14. These results indicate that the system operates near the diffraction-limited performance, demonstrating excellent optical precision and alignment.

### 4.2. Design Results of the Digital-Imaging Afocal Telescope System

**Figure 23:**
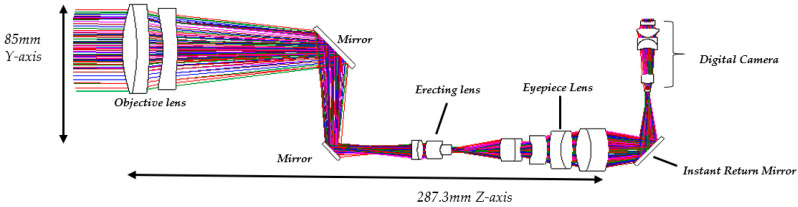
The overall system architecture of the digital-imaging afocal telescope system.

**Figure 24:**
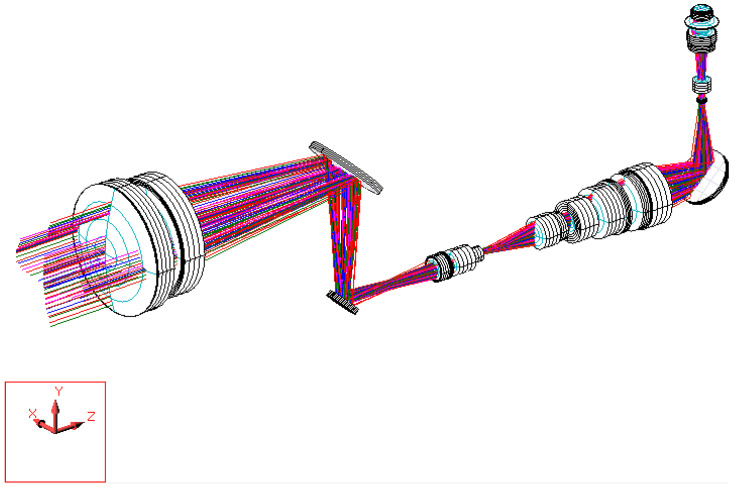
Three-dimensional diagram of the optical layout for the digital-imaging afocal telescope system.

**Figure 25:**
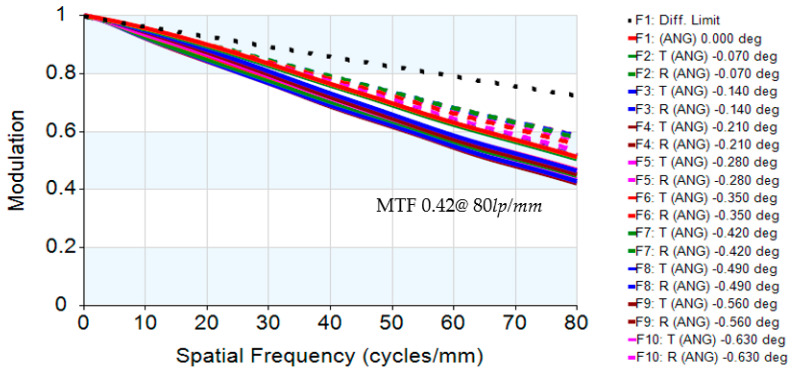
MTF of the optical layout for the digital-imaging afocal telescope system.

**Figure 26:**
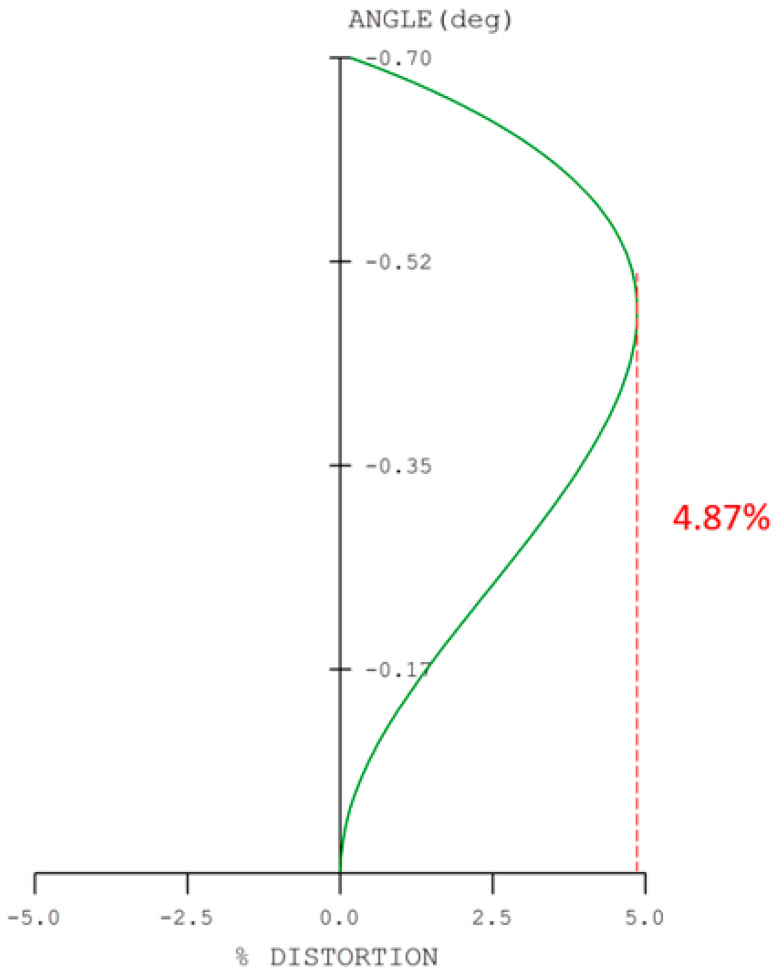
Distortion of the optical layout for the digital-imaging afocal telescope system.

**Figure 27:**
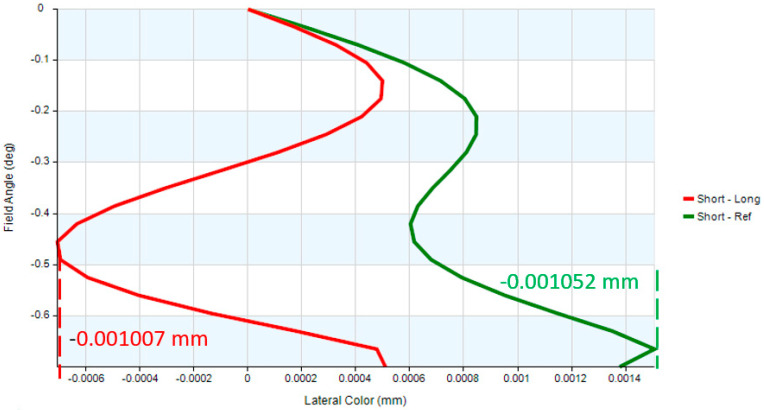
Lateral color of the optical layout for the digital-imaging afocal telescope system.

The digital-imaging afocal telescope system integrates a traditional telescope system with a digital-imaging unit, maintaining the original optical specifications without requiring further optimization. In this configuration, the aperture stop of each optical subsystem is positioned at its first surface, thereby placing the entrance pupil at the first optical surface. The overall system provides an exit pupil distance (eye relief) of 50 mm, and the total optical length is 287.3 mm, as shown in Figure 23. Final imaging performance achieves an MTF of 0.42 at 80 lp/mm in the worst field. The 3D diagram of the optical layout for the digital-imaging catadioptric telescope system is shown in Figure 24. The MTF plot is shown in Figure 25. The distortion is 4.87%, as shown in Figure 26. Figure 27 shows the lateral chromatic aberration at −1.007 µm (red) and 1.052 µm (green).

## 5. Conclusions

The optical layout of the digital-imaging afocal telescope system designed in this study combines an afocal imaging optical system with a digital camera system. This paper presents several key design features and contributions, summarized as follows:

I. The objective lens system has an effective focal length of 170 mm and a back focal length of 152.2 mm. The optical axis is defined along the *z*-axis. To reduce the overall length of the objective system along the *z*-axis, two mirrors are introduced within the objective lens system. The separation between the two mirrors is 52 mm, which shortens the back focal length along the *z*-axis to 100.2 mm. As a result, the overall length of the telescope system is significantly reduced, preventing an excessively long structure, as illustrated in Figure 22.
II. A movable mirror tilted at 45° is placed between the telescope and the digital camera. This mirror enables dual operating modes: direct visual observation by the human eye and digital image capture by the camera. By switching the mirror position, the system can selectively provide real-time visual observation or digital image recording.
III. In the proposed optical design, the aperture stop of the digital camera lens must be located at the front of the lens or on its first optical surface, such that the aperture coincides with the entrance pupil. Moreover, the aperture position must coincide with the exit pupil of the telescope, namely the eye-relief location. This position corresponds to the optimal viewing point for the human eye, where the observed image is both brightest and sharpest. In addition, the entrance pupil diameter and the incident half-field angle of the digital camera lens must match the exit pupil size and exit angle of the telescope to ensure efficient optical coupling.
IV. The image data captured by the digital camera can be transmitted to augmented reality (AR) glasses. In the past two years, we have published three related papers on AR glasses design [27,28,29]. Since AR glasses are capable of displaying digital camera images, the integration of the proposed optical system with AR glasses offers substantial commercial and entertainment potential. This system is well suited for visual observation applications such as bird watching, whale watching, and large-scale sporting events. Furthermore, it minimizes visual fatigue caused by prolonged observation through the eyepiece, thereby enhancing user comfort and operational convenience.
V. The telescope system consists of three optical subsystems: the objective lens, erecting lens, and eyepiece lens systems. These subsystems were independently designed and subsequently integrated to form the complete telescope system. Although the resulting system satisfies the afocal condition, residual aberrations remain. While these aberrations have negligible impact on human visual observation, they can slightly degrade the optical performance of the digital telescope system when combined with an independently designed digital camera. Therefore, with the telescope parameters fixed—along with the aperture position, aperture size, and focal length of the camera lens—the camera lens parameters were further optimized to compensate for the residual aberrations of the telescope, thereby improving the overall optical performance of the digital telescope system.

## Figures and Tables

**Table 1:** The preliminary specifications for the proposed afocal telescope system.

Objective Lens		Erecting Lens		Eyepiece Lens	
Entrance Pupil Diameter	40 mm	Focal Length	30 mm	Exit Pupil Diameter	2.5 mm
$\mathrm{focal}\;\mathrm{length}\;f_{o}^{\prime }$	176 mm	$\mathrm{Object}\;\mathrm{Distance}\;S_{o}$	−45 mm	$\mathrm{focal}\;\mathrm{length}\;f_{e}$	−22 mm
$\mathrm{Half}\;\mathrm{View}\;\mathrm{angle}\;\theta_{o}$	−0.7°	$\mathrm{Image}\;\mathrm{Distance}\;S_{i}$	90 mm	$\mathrm{Half}\;\mathrm{View}\;\mathrm{angle}\;\theta_{e}$	−11.061°
$\tan\theta_{o}$	−0.012	$\mathrm{Object-side}\;{NA}_{er}$	0.113	$\tan\theta_{e}$	−0.195
$\mathrm{Image}\;\mathrm{height}\;h_{1}$	−2.15 mm	$\mathrm{Image-side}\;{NA}_{er}\prime$	−0.056	$\mathrm{Image}\;\mathrm{height}\;h_{2}$	4.3 mm
$\mathrm{Image-side}\;{NA}_{o}\prime$	0.113	Transverse magnification	−2	$\mathrm{Object-side}\;{NA}_{e}$	−0.056

**Table 2:** Requirements for the optical imaging quality of the afocal telescope system.

Item	Design Target
RMS optical path difference (OPD)	<0.07 λ
|Optical distortion|	≤2%
Lateral color	<1 Airy Disk
Conic constant K	−100 < K < 100
The range of wavelengths	656 nm, 587 nm, 486 nm.

**Table 3:** Tolerance parameter range.

Type	Design RMS OPD Value	Design plus Tolerance RMS OPD Value
DLF (fringe)	1~8 fringes	1~8 fringes
DLT (mm)	>0.02 mm	0.01 mm~0.05 mm
DLN	>0.0001	0.0001~0.0005
DLV	>0.1%	0.1%~0.5%
CYD (fringe)	>1 λ	0.1 λ ~1 λ
CYN (fringe)	>1 λ	0.1 λ ~1 λ
TRX (arcmin)	>0.5 arc min	1 arc min~3 arc min
TRY (arcmin)	>1 arc min	1 arc min~3 arc min
BTY (arcmin)	>1 arc min	1 arc min~3 arc min
BTX (arcmin)	>1 arc min	1 arc min~3 arc min

**Table 4:** Specification of the Omni-Vision 5 Mega CMOS sensor.

Omni-Vision Sensor Specifications	
Manufacturer	Omni Vision
Product model	OV5695
Effective pixels	2631 × 1973 (5 Mega)
Pixel size	1.4 μm × 1.4 μm
Area length and width	3.684 mm × 2.763 mm
Diagonal	4.605 mm

**Table 5:** Optical design parameters of the objective lens.

Surface No.	Type	Y Radius	Thickness	Glass	Y-Semi Aperture	Conic Constant (K)	4th Order	6th Order		8th Order	10th Order
Object	Sphere	Infinity	Infinity								
1	Asphere	65.77	7.5	PSF67_SCHOTT	20	−0.22	−2.2 × 10^−7^	6.36 × 10^−11^		−3.01 × 10^−12^	
2	Sphere	−110	6.33	PSF8_SCHOTT	19.79						
3	Asphere	−244.19	7.14		19.35	64.73	−4.56 × 10^−7^	1.78 × 10^−9^	−4.27 × 10^−12^		6.661 × 10^−15^
4	Asphere	−54.65	7.5	POLEFINH	18.34	−6.4	−1.83 × 10^−6^	4.97 × 10^−9^	2.95 × 10^−12^		5.88 × 10^−15^
5	Asphere	−95.42	80.2		17.74	−26.83	3.22 × 10^−6^	4.72 × 10^−6^	1.95 × 10^−12^		5.2 × 10^−15^
6	Sphere	Infinity	0		13.14						
7	Sphere	Infinity	−52	Reflect	9.48						
8	Sphere	Infinity	0		5.37						
7	Sphere	Infinity	20	Reflect	4.18						
Image	Sphere	Infinity	0		2.15						

**Table 6:** The tolerance analysis of the objective lens.

FIELD	Design RMS OPD Value	Design plus Tolerance RMS OPD Value
0.0	0.0767	0.1018
0.1	0.0759	0.1013
0.2	0.0736	0.0997
0.3	0.0736	0.0997
0.4	0.0698	0.0971
0.5	0.0646	0.0937
0.6	0.0594	0.0899
0.7	0.0517	0.0860
0.8	0.0456	0.0827
0.9	0.0425	0.0825
1.0	0.0515	0.0879

**Table 7:** Optical design parameters of the erecting lens.

Surface (No.)	Surface Type	Y Radius	Thickness	Glass	Y-Semi Aperture	Conic Constant (K)	4th Order	6th Order	8th Order	10th Order
Object	Sphere	Infinity	20.69							
1 (Stop)	Sphere	−186.94	3	NLASF45_SCHOTT	2.36					
2	Sphere	13.74	3	NLAK34_SCHOTT	2.77					
3	Sphere	−14.18	1		3.10					
4	Asphere	11.27	7.76	PSF67_SCHOTT	3.24	−0.26	−1.8 × 10^−5^	−1 × 10^−6^	1.09 × 10^−7^	−4.4 × 10^−9^
5	Asphere	4.09	5.88	PLAF37_SCHOTT	2.45	−0.29	−6.13 × 10^−4^	−6.7 × 10^−7^	−2.48 × 10^−6^	
6	Sphere	7.40	24.68		2.16					
7	Sphere	14.69	8	NLASF40_SCHOTT	5.78					
8	Sphere	−147.69	3	NFK58_SCHOTT	4.98					
9	Sphere	12.94	2.99		4.37					
Image	Sphere	Infinity	0.0000		4.22					

**Table 8:** The tolerance analysis of the erecting lens.

Field	Design RMS OPD Value	Design plus Tolerance RMS OPD Value
0.0	0.0305	0.0466
0.1	0.0310	0.0472
0.2	0.0322	0.0490
0.3	0.0338	0.0516
0.4	0.0356	0.0545
0.5	0.0369	0.0571
0.6	0.0374	0.0589
0.7	0.0373	0.0600
0.8	0.0379	0.0619
0.9	0.0428	0.0694
1.0	0.0579	0.0897

**Table 9:** Optical design parameters of the eyepiece lens.

Surface (No.)	Surface Type	Y Radius	Thickness	Glass	Y Semi Aperture	Conic Constant (K)	4th Order	6th Order	8th Order	10th Order
Object	Sphere	Infinity	Infinity							
1 (Stop)	Sphere	Infinity	50		1.25					
2	Sphere	30	9.18	NSF66_SCHOTT	11.47					
3	Sphere	25.66	8	NPK52A_SCHOTT	10.54					
4	Asphere	−20.88	1.43		10.52	−4.13	6.27 × 10^−6^	2.36 × 10^−8^	−3.85 × 10^−11^	−1.99 × 10^−13^
5	Sphere	55.47	6.27	NLAK7_SCHOTT	9.71					
6	Sphere	−18.63	5.84	NSF57_SCHOTT	9.17					
7	Sphere	−39.85	1		8.48					
8	Sphere	25.24	7.85	PSF67_SCHOTT	7.29					
9	Sphere	8.25	3		4.45					
Image	Sphere	Infinity	0		4.25					

**Table 10:** The tolerance analysis of the eyepiece lens.

Field	Design RMS OPD Value	Design plus Tolerance RMS OPD Value
0.0	0.0068	0.0182
0.1	0.0099	0.0200
0.2	0.0155	0.0239
0.3	0.0212	0.0283
0.4	0.0271	0.0335
0.5	0.0342	0.0418
0.6	0.0425	0.0531
0.7	0.0515	0.0652
0.8	0.0590	0.0750
0.9	0.0644	0.0814
1.0	0.0649	0.0997

**Table 11:** Preliminary design specification for digital camera system.

**Half of image high**	2.30 mm
**Half of view angle**	11.06°
**EFL**	11.77 mm
**F-number**	4.71
**MTF (80 lp/mm)**	>0.5
**Lateral color**	<3.37 μm

**Table 12:** Optical design parameters of the digital camera system.

Surface (No.)	Surface Type	Y Radius	Thickness	Glass	Y Semi Aperture	Conic Constant (K)	4th Order	6th Order	8th Order	10th Order
Object	Sphere	Infinity	Infinity							
1 (Stop)	Asphere	29.75	1.15	NKZFS11_SCHOTT	1.25	100	1.82 × 10^−3^	2.55 × 10^−4^	−1.33 × 10^−4^	1.63 × 10^−5^
2	Asphere	11.37	3.19		1.36	−3.91	3.91 × 10^00^	3.76 × 10^−3^	6.12 × 10^−4^	−2.55 × 10^−4^
3	Asphere	23.82	4.8	NPK51_SCHOTT	2.23	−100	1.66 × 10^−3^	7.01 × 10^−5^	−3.27 × 10^−5^	3.00 × 10^−6^
4	Sphere	−12.59	13.23		2.76					
5	Sphere	−11.06	4.67	NLAK33A_SCHOTT	3.64					
6	Sphere	−5.53	0.8	SF2_SCHOTT	4.25					
7	Sphere	597.53	0.1		4.62					
8	Asphere	5.40	4.8	PLAK35_SCHOTT	5.26	−0.63	2.70 × 10^−4^	−1.98 × 10^−5^	5.69 × 10^−7^	−1.08 × 10^−8^
9	Sphere	−31.12	1.54		4.78					
10	Asphere	−33.61	1.64	PSF67_SCHOTT	3.37	83.02	−1.59 × 10^−3^	7.78 × 10^−4^	−6.97 × 10^−5^	2.37 × 10^−6^
11	Asphere	27.19	0.74		2.49	82.28	7.76 × 10^−3^	−1.92 × 10^−4^	1.98 × 10^−4^	−1.78 × 10^−5^
12	Sphere	Infinity	0.7	BK7_SCHOTT	2.48					
13	Sphere	Infinity	0.66		2.45					
Image	Sphere	Infinity	0		2.41					

**Table 13:** The tolerance analysis of the digital camera system.

	FIELD	Design RMS OPD Value	Design plus Tolerance RMS OPD Value
TAN	0.0	0.7206	0.6906
TAN	0.1	0.7076	0.6673
TAN	0.2	0.6435	0.5886
TAN	0.3	0.6180	0.5603
TAN	0.4	0.6382	0.5894
TAN	0.5	0.6461	0.6061
TAN	0.6	0.6429	0.5963
TAN	0.7	0.6340	0.5854
TAN	0.8	0.5942	0.5181
TAN	0.9	0.5552	0.4738
TAN	1.0	0.5662	0.4162
RAD	0.1	0.7189	0.6800
RAD	0.2	0.7091	0.6685
RAD	0.3	0.6900	0.6530
RAD	0.4	0.6889	0.6569
RAD	0.5	0.6901	0.6554
RAD	0.6	0.6887	0.6450
RAD	0.7	0.6884	0.6375
RAD	0.8	0.6855	0.6304
RAD	0.9	0.6879	0.6378
RAD	1.0	0.7026	0.6669

**Table 14:** RMS and Strehl ratio for the afocal telescope system.

	FRACT	DEG	RMS	STREHL
X Y	0.00 0.00	0.00 0.00	0.0438	0.927
X Y	0.00 0.10	0.00 −0.07	0.0413	0.935
X Y	0.00 0.20	0.00 −0.14	0.0407	0.937
X Y	0.00 0.30	0.00 −0.21	0.0414	0.935
X Y	0.00 0.40	0.00 −0.28	0.0405	0.937
X Y	0.00 0.50	0.00 −0.35	0.0417	0.934
X Y	0.00 0.60	0.00 −0.42	0.0403	0.938
X Y	0.00 0.70	0.00 −0.49	0.0399	0.939
X Y	0.00 0.80	0.00 −0.56	0.0383	0.944
X Y	0.00 0.90	0.00 −0.56	0.0355	0.951
X Y	0.00 1.00	0.00 −0.70	0.0474	0.915

## Data Availability

The original contributions presented in this study are included in the article. Further inquiries can be directed to the corresponding author.
